# Supplementary material for: Getting More Out of Biomedical Documents with GATE's Full Lifecycle Open Source Text Analytics
Source: PLoS Comput Biol. 2013 Feb 7;9(2):e1002854. doi: 10.1371/journal.pcbi.1002854 (PMC3567135; doi:10.1371/journal.pcbi.1002854)
Supplement: Dataset S1 — GATE software. Dataset S1 bundled with this paper contains a distribution of GATE (or see http://gate.ac.uk/download/). (TGZ) [file pcbi.1002854.s001.tgz › plos-gate/src/gate/resources/texts/news/BT-obs-13-May-2001.html]

Observer | Debt-ridden BT set to pay Bonfield £3m

**Debt-ridden BT set to pay Bonfield £3m**


**Jamie Doward, deputy business editor**  
**Sunday May 13, 2001**  
**The Observer**

Embattled BT chief executive Sir Peter Bonfield could earn £3 million this year, according to the small print in his contract agreed just days after new chairman Sir Christopher Bland took the reins at the telecoms giant.

The payout, potentially double what the City had believed the BT boss could receive, is bound to anger shareholders, who have been asked to subscribe to a £5.9 billion rights issue to help the company reduce its debts. On the back of the rights issue BT announced that investors would not receive a dividend.

Bonfield's new contract, signed on 4 May, states that he is entitled 'to receive an award in 2001 of 200 per cent of salary under BT's incentive share plan and 100 per cent of bonus (worth £481,000) under BT's deferred bonus plan'. Bonfield earns a salary of £820,000 which means, in all, he could end up pocketing more than £2.94m for running BT this year, the lion's share of which would come in the form of shares.

A BT spokesman said the package was dependent on a wide range of performance and budget targets being met.

The company is restructuring, demerging its wireless arm and selling off non-core assets in a bid to give it a new focus. On Friday BT's hopes of selling Yell, its directories division, suffered a setback when the Office of Fair Trading announced plans to impose price caps on the business. There are now fears that potential buyers, notably a consortium led by venture capital firm Apax Partners, will lose interest in Yell.

'If Sir Peter completes the restructuring programme over the next 18 months he will earn a lot of money. We want him to stay and this is a vote of confidence in him,' the spokesman said.

A complicated series of clauses suggests the £3m payout is largely dependent on Bonfield staying in his job beyond 31 December 2002, a date when he would be entitled to a further bonus of £820,000, providing the restructuring deadline has been met. Another clause states that Bonfield would not be due to receive his 2001 awards and bonus for three years, according to those who have seen the contract's small print.

Despite BT's efforts to snap golden handcuffs on Bonfield, many doubt whether he will still be in his job by the end of next year, potentially threatening the payout. Sources suggest Bonfield wants to stand down once BT demerges its wireless division. Preparation for the move has been going on for several months and insiders say it is now almost ready for its demerger.

The BT spokesman said the question of whether Bonfield would receive the bonuses if he left early was 'a matter for the remuneration committee'.

**Related articles**  
10.05.2001: BT bifurcates to appease shareholders  
09.05.01: BT sees profit at cutting edge lab  
07.05.01: BT prepares to bite the bullet  
**Useful links**  
BT  
BT Cellnet  
London stock exchange

Guardian Unlimited © Guardian Newspapers Limited 2001
